# Investigation of the Vitamin D Metabolite Ratio (VMR) as a Marker of Functional Vitamin D Deficiency: Findings from the SarcoPhAge Cohort

**DOI:** 10.3390/nu16193224

**Published:** 2024-09-24

**Authors:** Aurélie Ladang, Anne-Sophie Gendebien, Stéphanie Kovacs, Céline Demonceau, Charlotte Beaudart, Stéphanie Peeters, Majed S. Alokail, Nasser M. Al-Daghri, Caroline Le Goff, Jean-Yves Reginster, Olivier Bruyere, Etienne Cavalier

**Affiliations:** 1 Department of Clinical Chemistry, CIRM, CHU de Liège, University of Liège, 4000 Liège, Belgiumetienne.cavalier@chuliege.be (E.C.); 2 Research Unit in Public Health, Epidemiology and Health Economics, University of Liège, 4000 Liège, Belgiumolivier.bruyere@uliege.be (O.B.); 3 Clinical Pharmacology and Toxicology Research Unit (URPC), NARILIS, Department of Biomedical Sciences, University of Namur, 5000 Namur, Belgium; 4 Protein Research Chair, Biochemistry Department, College of Science, King Saud University, Riyadh 11451, Saudi Arabia; 5 Chair for Biomarkers of Chronic Diseases, Biochemistry Department, College of Science, King Saud University, Riyadh 11451, Saudi Arabia; 6 Department of Physical Activity and Rehabilitation, University of Liège, 4000 Liège, Belgium

**Keywords:** vitamin D metabolite ratio (VMR), functional vitamin D deficiency, parathyroid hormone (PTH), mortality prediction

## Abstract

Background: The vitamin D metabolite ratio (VMR) has recently been identified as a potentially better indicator of vitamin D deficiency than 25-hydroxyvitamin D (25(OH)D) alone. This study aims to validate these findings by demonstrating that VMR is more strongly correlated with parathyroid hormone (PTH) levels than 25(OH)D and 24,25-dihydroxyvitamin D (24,25(OH)_2_D). In addition, the study investigates VMR as a more effective predictor of mortality than 25(OH)D and 24,25(OH)_2_D. Methods: The SarcoPhAge cohort is a Belgian cohort of community-dwelling older adults. Levels of 25(OH)D and 24,25(OH)_2_D were measured in 204 serum samples collected at the second year of follow-up using liquid chromatography–tandem mass spectrometry (LC–MS/MS), and VMR was calculated using the formula: VMR = (24,25(OH)D/25(OH)D) × 100. Vitamin D deficiency cut-offs were defined at 25(OH)D < 20 ng/mL, 24,25(OH)_2_D < 1.2 ng/mL, or VMR < 4% according to previously proposed cut-offs. Participants were followed for up to 9 years. Results: A total of 35 individuals (17.2%) had 25(OH)D < 20 ng/mL, 40 individuals (19.6%) had 24,25(OH)_2_D < 1.2 ng/mL, and 14 individuals (7.0%) had VMR < 4%. All three markers, 25(OH)D, 24,25(OH)_2_D, and VMR, were independently associated with PTH levels, with VMR showing the strongest correlation (rho: −0.292; *p* < 0.0001). When categorized into quartiles, only 24,25(OH)2D and VMR showed significant increases in PTH levels across quartiles (*p* = 0.002 and *p* < 0.0001, respectively). When cut-offs for low vitamin D status were applied, patients with low VMR had the highest rate of all-cause mortality. However, in a Cox proportional hazard regression model, both low VMR profile and low 25(OH)D profile were risk factors for all-cause mortality. Conclusions: This study confirms that VMR is an efficient biomarker for assessing functional vitamin D deficiency.

## 1. Introduction

Vitamin D is a critical nutrient that plays an important role in maintaining bone, and its deficiency can lead to many adverse outcomes such as rickets or osteomalacia. Its importance extends beyond bone health to influence several physiological processes, including immune function, cardiovascular health, several cancers, diabetes, cognitive function, and muscle [1,2,3]. Despite large research and public health initiatives, worldwide prevalence of vitamin D deficiency remains high in many regions [1,4,5,6,7]. In addition, screening of the general population for vitamin D deficiency has shown some limitations in preventing outcomes [1]. Active screening for vitamin D deficiency at the population level, in the absence of a clinical presentation, does not appear to be justified, but screening and/or routine supplementation may be appropriate in high-risk populations, for example, older individuals in residential care and those with pigmented skin living in northerly latitudes [8,9]. Therefore, more effective diagnostic strategies for vitamin D deficiency are required.

The current standard for assessing vitamin D status involves measuring serum levels of 25-hydroxyvitamin D [25(OH)D]. Indeed, total 25(OH)D, the sum of 25(OH)D2 and 25(OH)D3, is usually considered the best indicator of vitamin D in the body, as observational and interventional studies have shown its ability to prevent cytoskeletal outcomes [10,11,12]. However, measuring 25(OH)D has significant limitations. First, some studies have shown that individuals with low 25(OH)D levels may not have the expected clinical signs of deficiency, such as bone mineralization defects or secondary hyperparathyroidism (elevated parathyroid hormone (PTH) levels) [13], suggesting that 25(OH)D is not a complete measure of vitamin D sufficiency. Second, 25(OH)D levels are differentially associated with bone mineral density, depending on factors such as ethnicity, geographical location and season. For example, black Americans, although having less 25(OH)D compared to white Americans, have a higher bone mineral density and a lower risk of fragility fractures [14,15]. In addition, although a reference method exists, some commercial methods have not been standardized yet. This situation increases the analytical variation between assays and the discrepancies between reported results [16].

Given these limitations, there has been increasing interest in alternative biomarkers based on other vitamin D forms that may provide a more comprehensive assessment of vitamin D status. One promising approach is the measurement of 24,25-dihydroxyvitamin D [24,25(OH)_2_D], the major catabolic product of 25(OH)D, together with the vitamin D metabolite ratio (VMR), defined as the ratio of 24,25(OH)_2_D to 25(OH)D multiplied by 100 [17]. This approach has several advantages over traditional 25(OH)D measurements. Indeed, we have previously shown in a large population of children that, for the same 25(OH)D concentration, some of them had already started to catabolize 25(OH)D and presented quantifiable 24.25(OH)2-vitamin D concentrations, whereas some other did not, revealing the potential personalization of an individual threshold for vitamin D [18]. Additional research has shown that VMR provides better information about vitamin D status and bone health than 25(OH)D levels alone. For example, individuals with a low VMR (<4%) often have higher PTH levels and markers of bone turnover, indicating functional vitamin D deficiency regardless of serum 25(OH)D concentrations [19]. This suggests that VMR may better identify individuals at risk of bone-related complications and other health problems associated with vitamin D deficiency than 25(OH)D alone. Furthermore, despite significantly lower 25(OH)D levels in African Americans, Berg et al. showed that the average VMR and bone health metrics were comparable to those of white participants [20]. In the context of population screening and prevention of outcomes, Herrmann et al. has found that a low VMR was associated with higher all-cause mortality, independent of 25(OH)D levels [19]. This supports the notion that VMR captures critical aspects of vitamin D metabolism that are not reflected by 25(OH)D measurements alone.

This study aims to confirm the utility of the VMR as a superior biomarker for the assessment of functional vitamin D deficiency in the SarcoPhAge cohort of community-dwelling older adults in Belgium [21]. Specifically, this study aims to (1) assess if VMR correlates better with PTH levels than 25(OH)D and 24,25(OH)_2_D and (2) assess the predictive value of VMR for long-term mortality compared to 25(OH)D and 24,25(OH)_2_D.

## 2. Methods

### 2.1. Population

The SarcoPhAge study is a prospective longitudinal cohort study designed to assess the health and functional consequences of sarcopenia in community-dwelling older adults [21]. The study was approved by the Ethics Committee of the University Teaching Hospital of Liège (number 2012/277) and all participants gave informed consent. Participants were recruited between June 2013 and June 2014 from various outpatient clinics in Liège, Belgium, including osteoporosis, geriatric, rheumatology, and rehabilitation centers, and through press advertisements. The inclusion criteria was community-dwelling individuals aged 65 years or older. A total of 534 subjects were enrolled, with a mean age of 73.5 ± 6.16 years, of whom 60.5% were women. Participants were followed up for up to nine years. Data collected included sociodemographic variables (age, sex, and BMI), clinical variables, and several health-related measures including mortality. The Strengthening the Reporting of Observational Studies in Epidemiology (STROBE) Statement was followed for this research [22].

### 2.2. Laboratory Measurements

Blood samples were taken from participants at the second-year follow-up visit. The samples were processed and stored under standardized conditions at −80 °C to ensure the accuracy and reliability of subsequent laboratory analyses.

The primary biomarkers measured in this study were 25(OH)D and 24,25(OH)_2_D. These measurements were made using a previously published liquid chromatography–tandem mass spectrometry (LC–MS/MS) method [23]. This method is certificated for the measurement of 25(OH)D by the Centers for Disease Control and Prevention (CDC) for its precision and accuracy based on the Vitamin D Standardization and Certification Program (VDSCP).

The VMR was calculated to assess the functional status of vitamin D metabolism in the participants. The VMR was calculated using the formula: VMR = $\frac{24,25\left(OH\right)2D}{25\left(OH\right)D}\times 100$.

### 2.3. Assessment of Outcomes

The primary outcome of this study was PTH levels, which were measured to assess the relationship between VMR and PTH, as elevated PTH is a known indicator of functional vitamin D deficiency. Blood samples for PTH measurement were taken at the same time as vitamin D status and analyzed using a Liaison 1-84 PTH immunoassay on Liaison XL analyzer (Diasorin, Italy). The association between PTH levels and different vitamin D metabolites, including VMR, 25(OH)D, and 24,25(OH)_2_D, was assessed.

Secondary outcomes included assessment of bone turnover markers (BTMs) and mortality. C-telopeptide collagen type 1 (CTXS) and propeptide N-terminal procollagen type 1 (PINP) were selected to evaluate bone turnover and both were measured on an Isys analyzer (IDS, England) according to the manufacturer’s instruction. Mortality was tracked over the follow-up period, with data collected through periodic interviews, medical records, and national death registries [24]. In case of confirmed death but unspecified date of death, death was arbitrarily set at the 1st of January 2024.

### 2.4. Statistical Analysis

Descriptive statistics were used to summarize baseline characteristics, including the distribution of vitamin D metabolites (25(OH)D, 24,25(OH)_2_D, and VMR) and outcomes (PTH, BTMs levels, and mortality). The prevalence of vitamin D deficiency was assessed using previously proposed cut-offs: 25(OH)D < 20 ng/mL [8], 24,25(OH)_2_D < 1.2 ng/mL, and VMR < 4% (arbitrarily derived from the DESIRE cohort) [19]. Statistical differences between normal profile and low profile groups were assessed with the independent Mann–Whitney test for continuous variables and with the Chi-squared test for dichotomous variables.

Spearman’s rank correlation coefficients were used to assess the associations between the three vitamin D markers (25(OH)D, 24,25(OH)_2_D, and VMR) and PTH levels. Participants were divided into quartiles according to their levels of 25(OH)D, 24,25(OH)_2_D, and VMR. The Kruskal–Wallis test was used to assess differences in PTH levels between these quartiles.

Cox proportional hazards regression models were used to assess the association between vitamin D markers and nine-year mortality. Categorical forms of vitamin D markers were analyzed. Kaplan–Meier survival curves and log-rank tests were used to compare mortality rates between groups with low and adequate vitamin D levels based on established cut-offs.

All statistical analyses were performed on Medcalc° version 22.021 (Medcalc software Ltd., Ostend, Belgium). Statistical significance was set at *p* < 0.05 for all tests.

## 3. Results

A total of 204 patients with complete assessment of 25(OH)D, 24,25(OH)_2_D, and PTH levels as well as mortality data were available at the second follow-up. A total of 49.5% of patients were male with a median age of 74.0 years old (IQR: 8.9) and a median body mass index (BMI) of 27.0 (IQR: 5.3). A total of 68.1% of the subjects were self-reporting oral vitamin D supplementation. During the 9-year follow-up period, 43 subjects died (21.1%) in relation to all-cause mortality. The median concentration was 30.9 ng/mL (IQR: 12.4) for 25(OH)D and 2.49 ng/mL (IQR: 1.85) for 24,25(OH)_2_D. A total of 5 subjects, one of whom died, had 24,25(OH)_2_D below our limit of quantification, disabling VMR calculation for these patients, and thus establishing the number of patients at 199 subjects with available VMR, among which 42 died.

According to cut-offs for vitamin D deficiency proposed by Hermann et al. (cut-offs that were arbitrarily fixed from the DESIRE cohort) [19], 35 individuals (17.2%) had 25(OH)D levels below 20 ng/mL, 40 individuals (19.6%) had 24,25(OH)_2_D levels below 1.2 ng/mL, and 14 individuals (7.0%) had VMR values below 4%. Of note, 5 individuals, in which 24.25(OH)_2_D was below the limit of quantification, were excluded from the VMR analysis since we were unable to calculate the ratio. Men were more likely to have low 25(OH)D or low 24,25(OH)_2_D (Table 1). No difference in terms of PTH or BTMs (PINP and CTXS) was observed between the normal or low profile groups, no matter which vitamin D parameter was assessed (Table 1).

**Figure 1:**
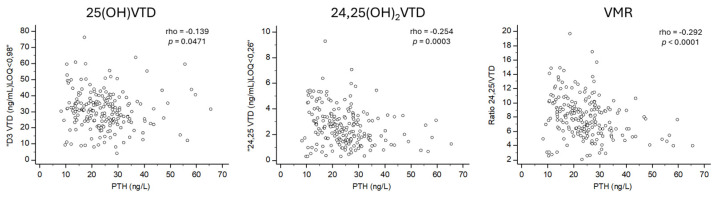
Correlation between PTH and vitamin D biomarkers.

**Figure 2:**
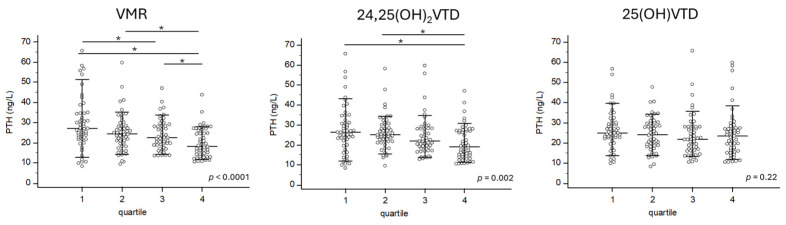
PTH is differentially expressed according to the VMR quartile but not according to the 25(OH)VTD quartile. The Kruskal–Wallis test was used to assess differences in PTH levels between these quartiles. Each subject is represented by a dot. * represent groups that are statistically different.

VMR showed the strongest correlation with PTH levels (rho = −0.292; *p* < 0.0001). 24,25(OH)_2_D also had a significant correlation with PTH levels (rho = −0.254; *p* = 0.0003), while 25(OH)D had a weaker but still significant correlation with PTH levels (rho = −0.139; *p* = 0.0471) (Figure 1). However, CTXS and PINP BTMs, were not associated with VMR, 24,25(OH)2D, or 25(OH)D. The Kruskal–Wallis test revealed significant increases in PTH levels across quartiles for VMR (*p* < 0.0001) and 24,25(OH)_2_D (*p* = 0.002). However, no significant changes in PTH levels were observed across quartiles for 25(OH)D (Figure 2).

**Figure 3:**
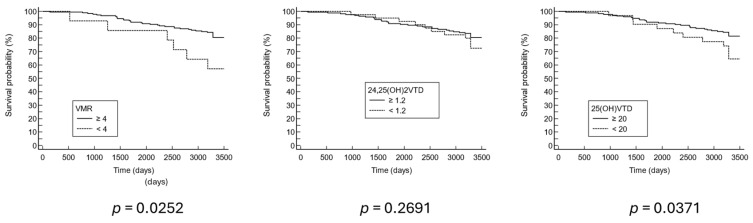
Nine-year Kaplan–Meier survival curve.

Survival analysis was performed to assess the predictive value of the vitamin D markers for nine-year mortality. Patients with a low VMR profile had the highest rate of all-cause mortality (42.9%) (Table 1). According to the Cox proportional hazards regression with age, sex, and BMI as other variables of the model, the hazard ratio was 3.74 for VMR (1.50–9.31, 95% confidence interval (CI)) and 2.41 (1.19–4.89, 95% CI) (Table 2). In addition, Kaplan–Meier survival curves indicated significant differences in mortality rates for both low VMR and low 25(OH)D profiles (Figure 3).

## 4. Discussion

This study suggests that the VMR may be a more effective marker of functional vitamin D deficiency than traditional markers such as 25(OH)D and 24,25(OH)_2_D. Indeed, VMR showed the strongest correlation with PTH levels, with significant increases in PTH across VMR quartiles. Furthermore, the low VMR profile participants had the highest rate of mortality, and more additional associations were discovered between 9-year all-cause mortality and low VMR or low 25(OH)D profiles. These findings suggest that VMR has potential as a more accurate biomarker for assessing vitamin D deficiency and its associated health risks.

Our results are consistent with the conclusions of previous studies that support a functional assessment of vitamin D status using the VMR and 24,25(OH)_2_D, suggesting that this approach provides superior diagnostic information compared to serum 25(OH)D levels alone. In the study by Herrmann et al. (2023), which included the DESIRE and LURIC cohorts, the authors evaluated the utility of a low vitamin D metabolite profile defined by 24,25(OH)_2_D < 1.2 ng/mL and VMR < 4% [19]. They found that this profile was associated with significantly higher parathyroid hormone (PTH) levels, accelerated bone turnover, and increased all-cause mortality, independent of serum 25(OH)D concentrations. These results support our findings that VMR is more strongly correlated with PTH levels than 25(OH)D and is a better predictor of long-term health outcomes, including mortality. Similar to our study, Herrmann et al. showed that individuals with low VMR had higher PTH levels, suggesting a more functional vitamin D deficiency, despite having 25(OH)D levels that would not traditionally be classified as deficient. Of note, both Herrmann’s and our LC–MS/MS method have shown very close results when compared together [25].

Additional strengths of VMR have been reported in other studies [17]. First, VMR remains stable despite significant fluctuations in vitamin D binding protein (VDBP) levels [26,27]. This stability underlines the potential of VMR as a reliable indicator of vitamin D status, unaffected by VDBP variability, which can confound vitamin D metabolites measurements. Furthermore, Ginsberg et al. found that lower VMR was significantly associated with a rapid decline in bone mineral density (BMD) and an increased fracture risk in older adults [28,29]. In contrast, 25(OH)D levels did not show a significant association with these outcomes. This supports the potential of VMR as a more sensitive marker of bone health, reflecting the metabolic balance of vitamin D more accurately than 25(OH)D alone.

The relationship between VMR and mortality, as demonstrated in both our study and Herrmann et al. [19], highlights the broader implications of VMR as an indicator of health outcomes beyond bone health. Although the direct link between VMR and PTH does not extend to mortality in our data, we propose that VMR captures critical aspects of vitamin D metabolism that are not solely reflected in bone-related biomarkers. Mortality is a multifactorial outcome, and the fact that low VMR was associated with increased all-cause mortality supports its potential utility as a global indicator of vitamin D deficiency [30]. The traditional reliance on 25(OH)D levels as the sole indicator of vitamin D status may be inadequate, as it does not take into account functional deficiencies that can affect bone health and all-cause mortality. The VMR may provide a more reliable diagnostic tool by more accurately reflecting vitamin D metabolism and its physiological effects [31,32,33]. Implementation of VMR in clinical practice could improve the identification of individuals at risk of vitamin D deficiency-related complications, allowing for more targeted and effective interventions. This approach could potentially lead to better patient outcomes through personalized treatment plans based on a comprehensive understanding of an individual’s vitamin D status [17]. However, further research is needed to establish more direct links between VMR and clinical outcomes, including bone fractures and disease-specific mortality. Interventional studies will be particularly important to validate whether targeting VMR can improve health outcomes more effectively than traditional vitamin D measures such as 25(OH)D. Additionally, the cost-effectiveness of this approach needs to be further investigated.

This study has several strengths, including the use of a well-defined cohort of community-dwelling older adults, the application of rigorous measurement techniques using liquid chromatography–tandem mass spectrometry (LC–MS/MS), and a long follow-up period of up to nine years. However, there are limitations. The study population, although well characterized, is limited to a relatively small number of older adults and to a specific European region, which may affect the generalizability of the findings to other populations. Potential confounders, such as variations in diet or vitamin D supplementation, physical activity, and sunlight exposure, were not considered. Furthermore, categorization of the population regarding vitamin D is based on arbitrary cut-offs previously proposed based on a single cohort. These cut-offs might bias a part of the results since the low VMR group is very reduced. The observational nature of the study precludes the establishment of causality between VMR and health outcomes, and additional interventional studies are required to properly validate cut-offs for functional vitamin D deficiency.

Based on the findings of this study and others, future research should focus on validating the clinical utility of VMR in larger and more diverse populations and especially in a population without vitamin D supplementation. In particular, randomized controlled trials are needed to assess whether interventions based on VMR measurements can lead to better health outcomes than traditional approaches using 25(OH)D levels alone, as these kinds of approaches have led so far to conflicting data [34,35,36,37]. In particular, criteria for functional vitamin D deficiency failed to identify subjects under vitamin D supplementation in a randomized controlled trial of hypertensive patients [38]. In addition, research into the biological mechanisms underlying the relationship between VMR, vitamin D metabolism, and health outcomes could provide further insights into the role of vitamin D in different physiological processes [17].

## 5. Conclusions

In conclusion, the VMR appears to be a more accurate and reliable marker for assessing functional vitamin D deficiency than traditional markers such as 25(OH)D and 24,25(OH)_2_D. The results of this study, supported by previous research, suggest that VMR better reflects vitamin D status and its associated health risks, including bone health and mortality. The implementation of VMR in clinical practice could improve the diagnosis and management of vitamin D deficiency, leading to improved patient outcomes. Further research is needed to validate these findings and explore the wider clinical applications of VMR.

## Figures and Tables

**Table 1:** Description of the cohort according to various cut-offs for functional vitamin D deficiency. Results are expressed as median (IQR). The statistical difference between the normal profile and low profile participants were assessed with the independent Mann–Whitney test for continuous variables and with the Chi-squared test for dichotomous variables. Statistically different results are highlighted in bold.

	VMR			24,25(OH)_2_D			25(OH)D		
	Normal	Low (≤4)	*p*-Value	Normal	Low (≤1.2 ng/mL)	*p*-Value	Normal	Low (≤20 ng/mL)	*p*-Value
n	185	14		164	40		173	31	
M/W	89/96	9/5	0.2442	**75/89**	**26/13**	**0.0084**	**81/92**	**20/10**	**0.0085**
Age (years)	74.2 (9.4)	71.7 (7.8)	0.3297	74.3 (9.2)	72.0 (6.7)	0.3636	74.2 (9.0)	72.3 (7.3)	0.5643
BMI (kg/m^2^)	26.7 (5.3)	28.0 (7.4)	0.3681	26.4 (5.3)	28.2 (5.4)	0.0599	26.7 (5.1)	28.4 (7.8)	0.1493
PTH (ng/L)	23.8 (10.8)	24.2 (19.1)	0.6842	23.2 (10.9)	26.6 (12)	0.1719	23.6 (11.3)	24.9 (10.0)	0.5226
CTXS (ng/L)	252.8 (244.7)	223.7 (459.4)	0.6587	251.0 (259.8)	223.7 (244.6)	0.6864	252.8 (263.6)	209.0 (227.6)	0.3595
PINP (ng/mL)	43.2 (28)	36.2 (39.7)	0.5347	44.0 (30)	39.9 (25.7)	0.2633	43.2 (29.2)	42.8 (21.5)	0.4972
Death at 9 years	**36**	**6**	**0.0391**	32	11	0.2261	**32**	**11**	**0.0332**

**Table 2:** Cox proportional-hazards regression. Statistically different results are highlighted in bold in tables.

	VMR		24,25(OH)2VTD		25(OH)VTD	
	OR (95% CI)	*p*-Value	OR (95% CI)	*p*-Value	OR (95% CI)	*p*-Value
Age (years)	**1.11 (1.05–1.16)**	**0.0001**	**1.09 (1.04–1.14)**	**0.0003**	**1.10 (1.04–1.15)**	**0.0002**
BMI (kg/m^2^)	0.94 (0.88–1.01)	0.0854	**0.92 (0.86–0.99)**	**0.0362**	**0.92 (0.86–0.99)**	**0.0272**
Sex	1.75 (0.91–3.35)	0.0930	1.85 (0.97–3.54)	0.0629	1.84 (0.97–3.52)	0.0626
Low profile	**3.74 (1.50–9.31)**	**0.0046**	1.68 (0.82–3.44)	0.1558	**2.41 (1.19–4.89)**	**0.0142**

## Data Availability

The raw data can be obtained on request from the corresponding author.
